# Machine learning discriminates a movement disorder in a zebrafish model of Parkinson's disease

**DOI:** 10.1242/dmm.045815

**Published:** 2020-10-16

**Authors:** Gideon L. Hughes, Michael A. Lones, Matthew Bedder, Peter D. Currie, Stephen L. Smith, Mary Elizabeth Pownall

**Affiliations:** 1 Department of Biology, University of York, York YO10 5DD, UK; 2 School of Mathematical and Computer Sciences, Heriot-Watt University, Edinburgh EH14 4AS, UK; 3 Australian Regenerative Medicine Institute, Monash University, Victoria 3800, Australia; 4 York Biomedical Research Institute, University of York, York YO10 5DD, UK; 5 Department of Electronic Engineering, University of York, York YO10 5DD, UK

**Keywords:** DJ-1, PARK7, Artificial intelligence, Gene targeting, Video capture, Parkinson's disease

## Abstract

Animal models of human disease provide an *in vivo* system that can reveal molecular mechanisms by which mutations cause pathology, and, moreover, have the potential to provide a valuable tool for drug development. Here, we have developed a zebrafish model of Parkinson's disease (PD) together with a novel method to screen for movement disorders in adult fish, pioneering a more efficient drug-testing route. Mutation of the *PARK7* gene (which encodes DJ-1) is known to cause monogenic autosomal recessive PD in humans, and, using CRISPR/Cas9 gene editing, we generated a Dj-1 loss-of-function zebrafish with molecular hallmarks of PD. To establish whether there is a human-relevant parkinsonian phenotype in our model, we adapted proven tools used to diagnose PD in clinics and developed a novel and unbiased computational method to classify movement disorders in adult zebrafish. Using high-resolution video capture and machine learning, we extracted novel features of movement from continuous data streams and used an evolutionary algorithm to classify parkinsonian fish. This method will be widely applicable for assessing zebrafish models of human motor diseases and provide a valuable asset for the therapeutics pipeline. In addition, interrogation of RNA-seq data indicate metabolic reprogramming of brains in the absence of Dj-1, adding to growing evidence that disruption of bioenergetics is a key feature of neurodegeneration.

This article has an associated First Person interview with the first author of the paper.

## Abstract (precis)

**Summary:** Using computational analyses to harness artificial intelligence, we have tested a genetic model of Parkinson's disease and reveal a distinct movement phenotype in adult zebrafish lacking Dj-1.

## INTRODUCTION

Parkinson's disease (PD) is common, affecting about 1% of the population over 60 (Tysnes and Storstein, 2017), and it has no cure. The incidence of PD increases with age; therefore, this statistic is likely to become worse owing to an ageing population. Most cases of PD are not inherited, arising sporadically; however, several monogenic forms of PD have been identified (reviewed in Hernandez et al., 2016). Studying the genes disrupted in patients with inherited forms of PD has been instrumental for progress in understanding the molecular basis of the disease (Clark et al., 2006; Clements et al., 2006; Shimura et al., 2000).

A valuable approach to understanding mechanisms by which gene mutations can result in human pathogenesis relies on *in vivo* experiments using animal models. Invertebrate models such as *Caenorhabditis* *elegans* (Ved et al., 2005) and *Drosophila* (Lu and Vogel, 2009) provide important platforms for studying neurodegenerative diseases in the context of a complete nervous system; however, the genetically tractable vertebrate *Danio rerio* shares more features of neuroanatomy with humans. Retrograde tracing has been used to identify dopaminergic (DA) neurons in zebrafish that project from the ventral diencephalon to the ventral telencephalon (Rink and Wullimann, 2001). These neurons are sensitive to the neurotoxin 1-methyl-4-phenyl-1,2,3,6-tetrahydropyridine (MPTP) (known to induce PD in humans) and considered analogous to the ascending midbrain DA neurons of the mammalian nigrostriatal pathway. In addition to anatomical similarities, vertebrate genomes share a high level of conservation such that orthologues of PD genes have been identified and manipulated in zebrafish (Anichtchik et al., 2008; Bretaud et al., 2007; Flinn et al., 2009; Keatinge et al., 2015; Zhao et al., 2012). However, most of the zebrafish models of PD have used transient post-transcriptional gene inhibition protocols focusing on the analysis of larval DA neurons (Bretaud et al., 2007; Flinn et al., 2009; Zhao et al., 2012) and swimming behaviour (Flinn et al., 2009; Zhao et al., 2012). In this study, we have developed an adult zebrafish model of PD that presents movement disorders typical of human patients.

Loss-of-function mutations in *PARK7* cause a rare form of early-onset PD (Bonifati et al., 2003). *PARK7* codes for the protein DJ-1, which has multiple roles in protecting cells from toxic protein aggregation and oxidative stress, and loss of these functions contributes to the presentation of parkinsonian pathology. DJ-1 protein is a member of a deeply conserved DJ-1/ThiJ/PfpI superfamily that includes related proteins from human to bacteria and archae (Bandyopadhyay and Cookson, 2004). Although these homologues share a core structure, their diverse functions include acting as kinases, proteases and chaperones. In humans, DJ-1 has been found to prevent the aggregation of α-synuclein (Zondler et al., 2014), regulate transcription of oxidative stress response genes (Xu et al., 2005) and maintain mitochondrial function (Thomas et al., 2011; Winklhofer and Haass, 2010). High levels of oxidative stress in DA neurons are characteristic of PD patients, so it is interesting that DJ-1 itself is activated by the oxidation of a specific cysteine residue (C106) that is essential for its translocation to the mitochondria and neuroprotective function (Canet-Avilés et al., 2004; Wilson, 2011). Structural biology (reviewed in Cookson, 2003) and *Drosophila* genetics (Oswald et al., 2018) have been particularly informative about the biochemistry and function of DJ-1, as a stable homodimer that acts as a redox sensor in neurons.

The advent of simple and effective gene editing in vertebrates means that it is feasible to disrupt orthologues of known disease genes to model human disease; to this end we have created a null mutation in *dj-1* (also known as *park7*) in zebrafish. In order to assess whether this mutant provides a good animal model of human disease, we have developed a novel method to discern recognisable traits of the condition, which will ultimately facilitate its use in developing treatments for improvement of symptoms. Neurodegeneration in PD is characterised by bradykinesia, resting tremor, rigidity and postural instability (Jankovic, 2008); of these, bradykinesia is a key indicative feature (Postuma et al., 2015). Recently, medical diagnosis of bradykinesia has been facilitated with a system called PD-Monitor that employs an evolutionary algorithm (EA) (a form of artificial intelligence or machine learning) to optimise predictive models capable of recognising bradykinesia from finger-tapping tasks (Gao et al., 2018). EAs can diagnose PD in humans with high accuracy from data collected using tracking sensors on the thumb and finger to extract movement data from a finger-tapping exercise performed by PD patients and healthy age-matched controls. The movement data were used to train an EA that evolved classifiers with diagnostic accuracies of 80-90% (Lones et al., 2013). Here, we report how we have adapted these methods to assess movement in a zebrafish model of PD using a simple video setup and a computational platform that could be widely used for assessing motor impairment phenotypes in zebrafish.

We have harnessed two powerful tools: gene editing to disrupt *dj-1*, generating an adult model of PD in zebrafish, and machine learning to evolve classifiers that discriminate movement data from our model. We find that training EAs with a continuous data stream mitigates bias, allows the computation of more wide-ranging features and can discriminate PD models from control zebrafish. We report a bradykinesia-like movement disorder in this model of PD, as well as an RNA-sequencing (RNA-seq) analysis that indicates metabolic reprogramming in the absence of DJ-1. This novel and simple platform for discerning movement phenotypes has the potential to make important contributions to future drug development for movement disorders.

## RESULTS

### Gene targeting of *dj-1* results in a null allele and loss of DA neurons

**Fig. 1.**
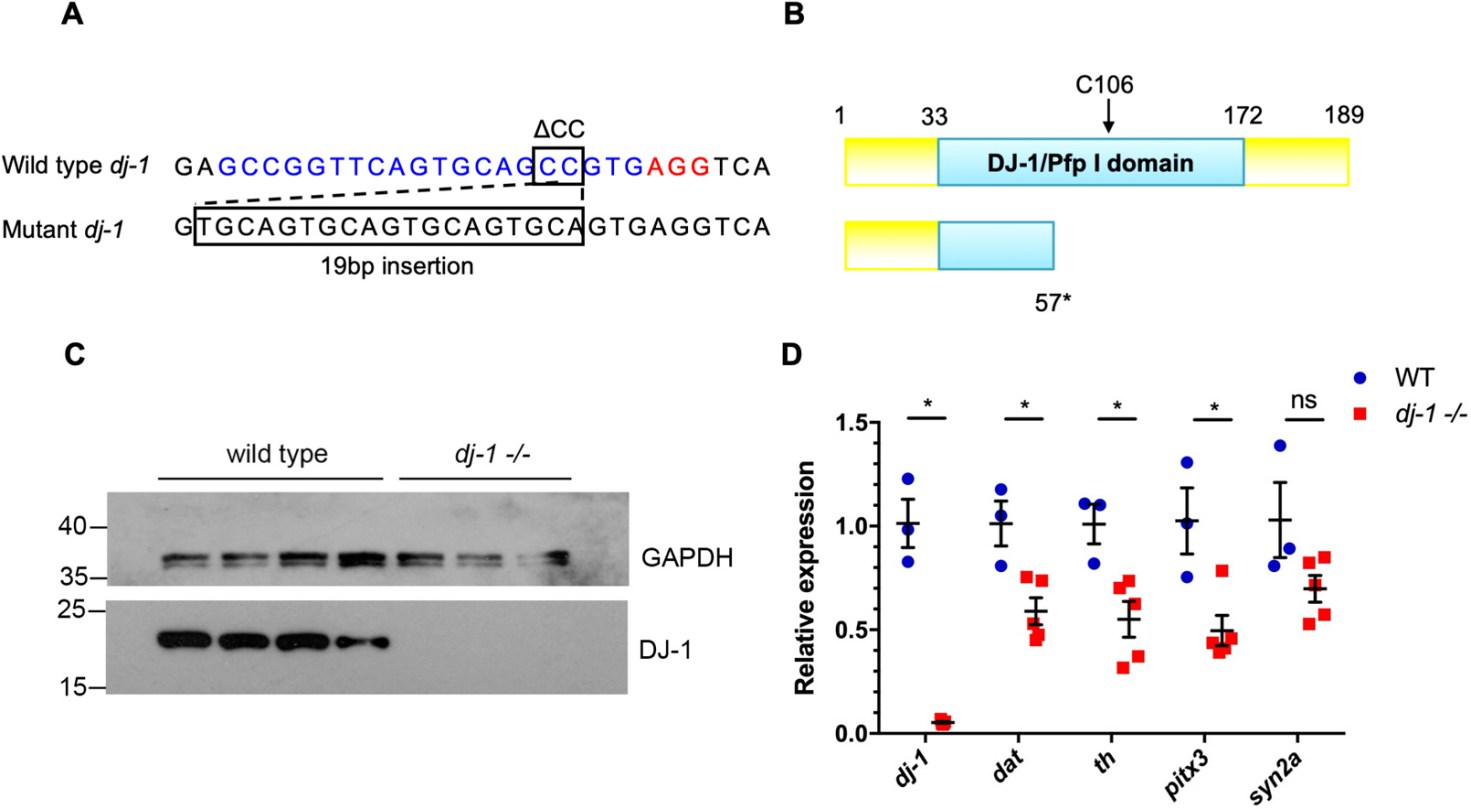
**The zebrafish** ***dj-1*^−/−^ mutation is a genetic null.** (A) Wild-type *dj-1* target sequence in the zebrafish genome (top). The 20 bp target sequence (blue) is directly upstream of a 3 bp protospacer adjacent motif (PAM) site (red). A 2 bp deletion (ΔCC) followed by a 19 bp insertion in the target sequence causes a frameshift mutation in *dj-1* (bottom). (B) Comparison of the protein structure for wild-type Dj-1 (top), with essential residue C106 indicated, and the predicted mutant protein truncated at residue 57 (bottom). (C) Western blot analysis of Dj-1 protein expression in the brains of wild-type adult zebrafish (lanes 1-4) and their *dj-1*^−/−^ mutant siblings (lanes 5-7). Gapdh was used as a loading control. (D) qRT-PCR analysis (single replicate) comparing gene expression in brains extracted from wild-type adult zebrafish (*n*=3, biological replicates) and their *dj-1*^−/−^ mutant siblings (*n*=5) at 16 weeks post-fertilisation (wpf). Target gene *dj-1* was analysed alongside DA neuron markers *dopamine transporter* (*dat*), *tyrosine hydroxylase* (*th*) and *pituitary homeobox 3* (*pitx3*); *synapsin IIa* (*syn2a*) acted as a general synapse marker. Student’s *t*-tests (two-tailed, unpaired) were used to compare the dCt values for *dj-1*^−/−^ and wild-type samples. Data are mean±s.e.m., *P<0.05; ns, not significant.

A BLAST search was used to identify any orthologues of *PARK7* (*DJ-1*) in the zebrafish and to compare the amino acid sequences of the encoded proteins. A single *DJ-1* orthologue, containing six exons, was identified in zebrafish on chromosome 11 (ENSDARG00000116835). The 189-amino acid protein encoded by *dj-1* in zebrafish shared 83% sequence identity with human DJ-1. Heterozygous *dj-1* mutants were identified in the F1 generation with a 2 bp deletion followed by a 19 bp insertion in exon 2 at position 200 in *dj-1* (NM_001005938; chr11:41459837) (Fig. 1A). This resulted in a frameshift and a premature stop codon at nucleotide position 227. The predicted Dj-1 protein translated from the mutant transcript would be 56 amino acids in length, missing the essential C106 residue (Fig. 1B). The heterozygous *dj-1* zebrafish carrying this mutation were in-crossed to create progeny (F2 generation), with 25% of the animals homozygous for the mutation. At maturity (16 weeks), western blot analysis using an antibody known to recognise zebrafish Dj-1 (Bai et al., 2006) revealed a complete loss of the ∼20 kDa Dj-1 protein that is detectable in wild-type but not in *dj-1^−/−^* mutant brains (Fig. 1C). Quantitative reverse transcription PCR (qRT-PCR) analysis was carried out on RNA extracted from the brains of five *dj-1^−/−^* mutants and three wild-type siblings for gene expression analyses. qRT-PCR showed a 95% loss of *dj-1* expression in the *dj-1^−/−^* brains, indicating nonsense-mediated decay of the transcript (Fig. 1D). Genes known to be expressed in (DA) neurons – *dopamine transporter* (*dat*; also known as *slc6a3*), *tyrosine hydroxylase* (*th*) and *paired-like homeodomain 3* (*pitx3*) – were also found to be downregulated in *dj-1^−/−^* mutant brains compared to their wild-type siblings. *dat* and *th* expression were assessed as both are markers of DA neurons; *dat* is specific to DA neurons, whereas *th* is expressed in all catecholaminergic neurons (Holzschuh et al., 2001). *pitx3* is a transcription factor involved in the development and maintenance of DA neurons (Filippi et al., 2007). We conclude that our targeting of *dj-1* has resulted in a line of a genetic null zebrafish.

**Fig. 2.**
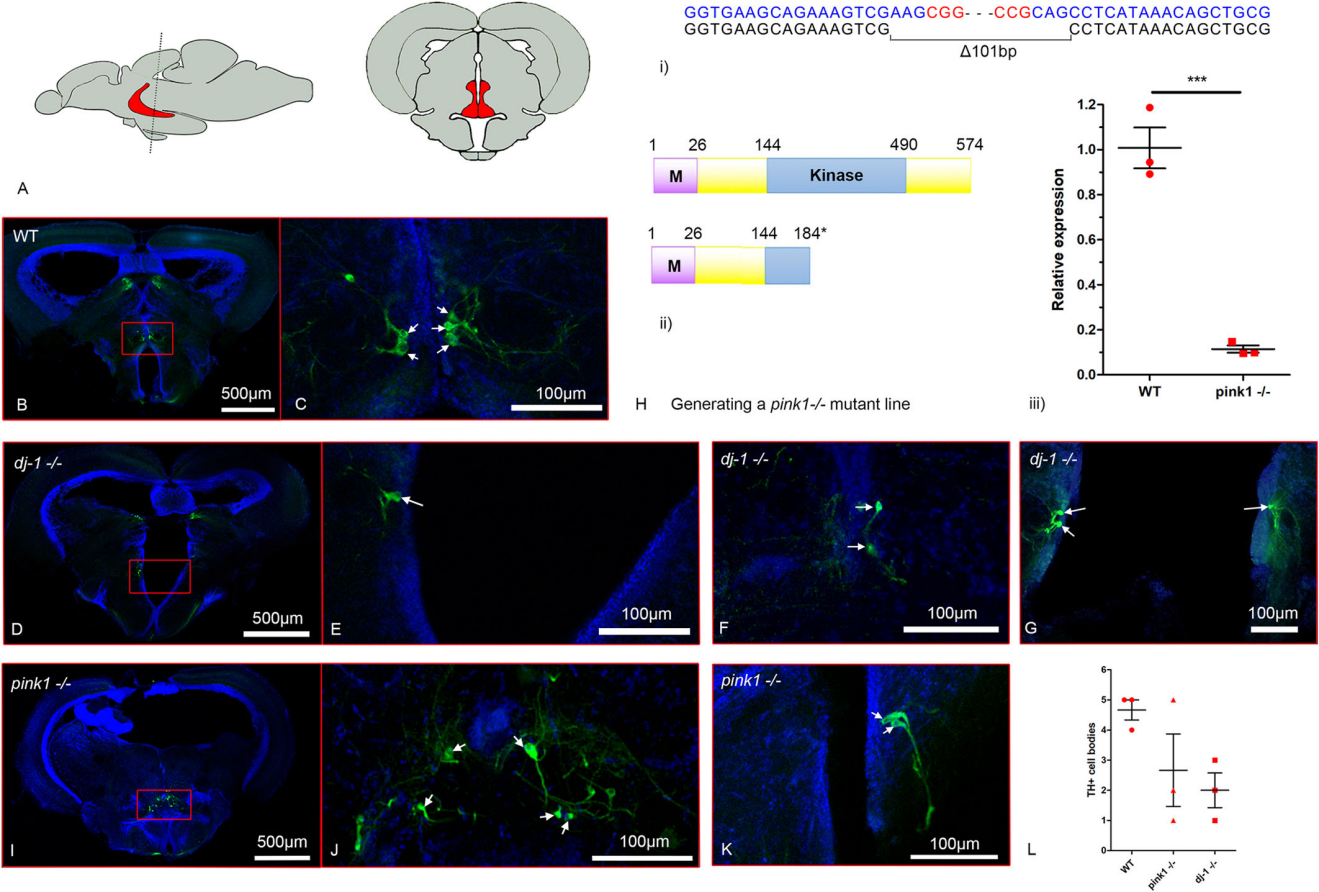
**Reduction of dopaminergic neurons in the posterior tuberculum of PD zebrafish.** Immunohistochemical detection of Tyrosine hydroxylase (Th) in *dj-1^−/−^* and *pink1^−/−^* brains. A section through the posterior tuberculum (pT) was identified based on the shape of the brain according to Wulliman et al. (1996). The large pear-shaped Th-positive cells located next to the ventricle in the pT have previously been identified as part of the zebrafish dopaminergic system projecting to the striatum (Rink and Wullimann, 2001). Therefore, the pear-shaped Th-positive cells identified in the periventricular pT location were counted. (A) Lateral view of the adult zebrafish brain (left) and cross-section through the adult zebrafish brain (right). Highlighted in red is the pT. (B-G,I-K) Immunofluorescently labelled Th-positive cells (green) in the pT of wild-type, *dj-1^−/−^* and *pink1^−/−^* zebrafish (*n*=3 biological replicates) at 12 months post-fertilisation (mpf). Hoechst staining of nuclei is in blue. Arrows indicate the cell bodies of the paraventricular DA neurons. (B) Th-positive cells in a section through the pT (red box) of a wild-type brain. (C) A close-up of the Th-positive cells in the pT from A. (D) Th-positive cells in a section through the pT (red box) of a *dj-1^−/−^* brain. (E) A close-up of the Th-positive cells in the pT from D. (F,G) Close-ups of Th-positive cells in the pT of two further *dj-1^−/−^* brains. (H)(i) CRISPR/Cas9 target sequences (blue), PAM sites (red) and the 101 bp deletion generated in exon 2 of *pink1* (at position 591 of NM_001008628)*.* (ii) Wild-type Pink1 (above) and the truncated Pink1 protein (below) predicted in the *pink1* mutant. (iii) qRT-PCR analysis comparing *pink1* expression in brains extracted from *pink1^−/−^* zebrafish (*n*=4, biological replicates) and their wild-type siblings (*n*=3) at 16 wpf. Student’s *t*-tests (two-tailed, unpaired) were used to compare the dCt values for *pink1*^−/−^ and wild-type samples. Data are mean±s.e.m., ***P<0.001. (I) Th-positive cells in a section through the pT (red box) of a *pink1^−/−^* brain. (J) A close-up of the Th-positive in the pT from I. (K) A close-up of the Th-positive cells in the pT of a further *pink1*^−/−^ brain. (L) Counts of Th-positive cell bodies seen in the pT (single 100 µm section) for wild-type, *dj-1^−/−^* and *pink1*^−/−^ zebrafish (*n*=3 biological replicates) at 12 mpf.

A *PTEN-induced kinase 1* null (*pink1^−/−^*) mutant line was generated by CRISPR/Cas9 targeting of the *PINK1* orthologue in zebrafish with two guide RNAs (gRNAs) producing a 101 bp deletion in exon 2 of *pink1* (at position 591 of NM_001008628) (Fig. 2H). The predicted Pink1 protein translated from the mutant transcript would be 183 amino acids in length, losing the majority of the kinase domain, and qRT-PCR analysis of RNA extracted from *pink1^−/−^* (*n*=4) and wild-type sibling (*n*=3) adult brains revealed a ∼90% loss of *pink1* expression, indicative of nonsense-mediated decay of the mRNA. An immunohistochemical analysis was used to detect DA neurons (Fig. 2); three brains were dissected from wild-type, *dj-1^−/−^* and *pink1^−/−^* zebrafish at 12 months post-fertilisation (mpf), and 100 µm sections were cut with a vibratome through the posterior tuberculum (pT) in the diencephalon. In zebrafish, the pT includes a subset of DA neurons that project to the subpallium; these cells are considered homologous to the anterior-most ascending mesodiencephalic DA neurons in mammals (groups A8-A10), which are affected in PD (Rink and Wullimann, 2002, 2001). To investigate whether these cells are impacted in our zebrafish models of PD, we used immunofluorescence to detect the protein Tyrosine hydroxylase (Th), an enzyme required to produce catecholamines including dopamine. Our method is informed by the work of Matsui and Sugie (2017) and Rink and Wullimann (2001) to identify and count these neurons in zebrafish. The brains were fixed and embedded in agarose blocks for vibratome sectioning before processing by immunohistochemistry for Th immunoreactivity and imaging by confocal microscopy. Cell bodies in the pT positive for Th that have a pear-shaped appearance and a periventricular position were identified as pT DA neurons and counted in each of the sections (Fig. 2B-G,I-K). Fig. 2L indicates the mean number of pT DA neurons identified for each genotype. In the wild-type brain, a range of four to five of these cells was identified, consistent with the retrograde tracing study by Rink and Wullimann (2001), while the number of pT DA neurons identified in the *dj-1^−/−^* and *pink1^−/−^* brains ranged from one to three and one to five, respectively, suggesting a reduction of these neurons in these genotypes, as shown previously for *pink1*^−/−^ (Flinn et al., 2013).

### A simple and effective method for measuring micro-movement in zebrafish

A progressive loss of DA neurons is characteristic of PD in humans (Damier et al., 1999); however, to establish whether our *dj-1^−/−^* null zebrafish provide a bone fide animal model of PD we wanted to test whether the fish share a dyskinesia phenotype. To do this, we developed a new and simple method to measure features of movement. In contrast to other fish-tracking programs, our analysis extracts movement data from the adult tail bending along its whole axis while accelerating, decelerating and turning. These data are collected as a continuous feed and converted by bespoke software into data suitable for use with novel computational methods to test whether our zebrafish model shows micro-movement features identified in human patients with PD.

**Fig. 3.**
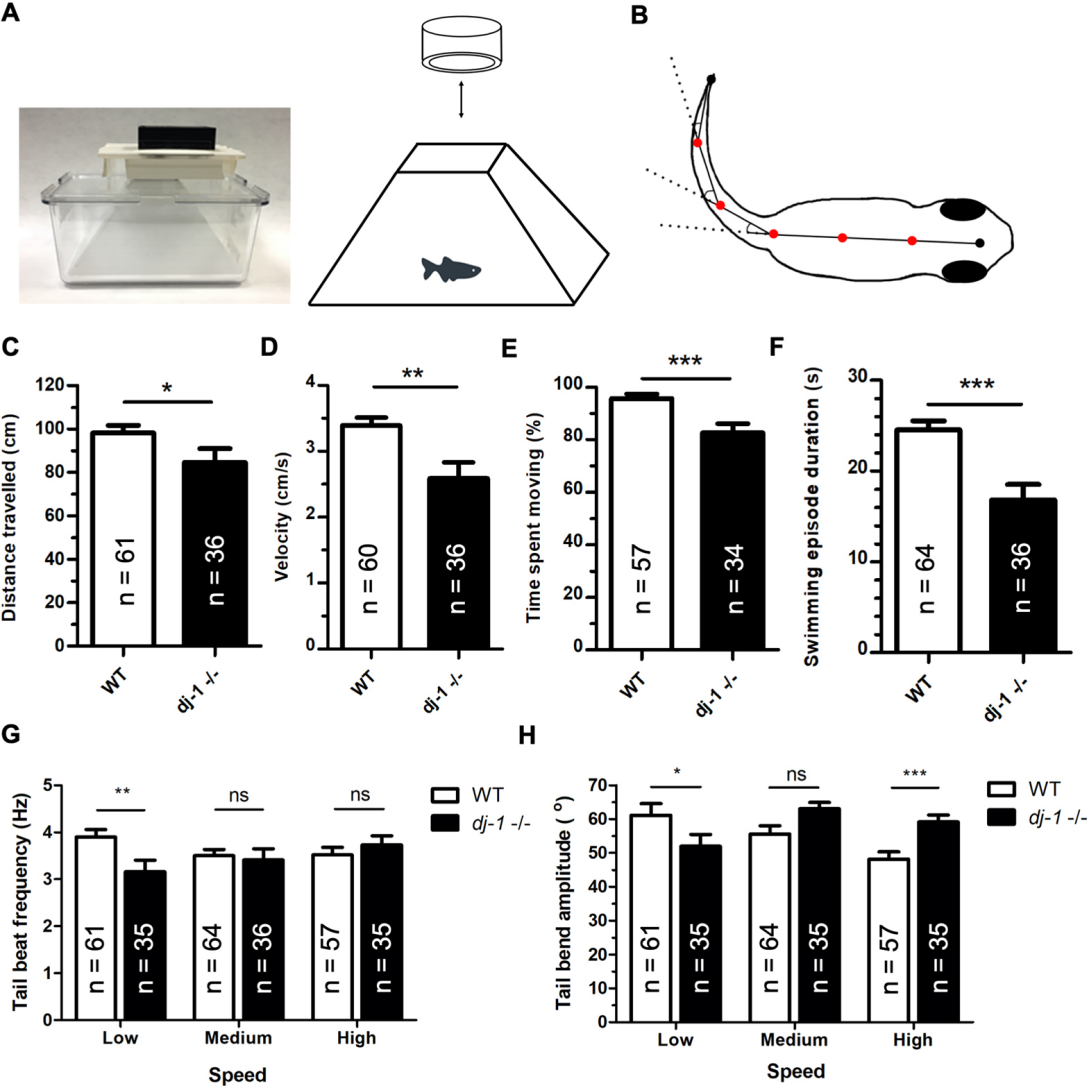
**Analysis of extracted features reveals distinct movement in *dj-1^−/−^* zebrafish.** (A) A photograph of the frustum insert designed to fit an Aquatics Habitat mating tank with a GoPro camera attached (left), providing a simple system for accurate, high-resolution video capture of adult zebrafish movement. A diagram of the fish inside the frustrum insert, recorded from above using the GoPro camera (right). (B) A diagram of the angles measured along the zebrafish trace, at the five vertices (red dots), when there is a bend in the tail. *x* and *y* coordinates were measured for the vertices and endpoints. Measurements were recorded at 100 frames/s from the video input, allowing analysis of selected features of movement. (C-H) The features of movement compared between *dj-1*^−/−^ and wild type (WT) at 12 wpf including distance travelled (C), velocity (D), percentage of time spent moving (E), mean duration of a swimming episode (F), tail beat frequency at low, medium and high swimming speeds (G), and tail bend amplitude at low, medium and high swimming speeds (H) (single replicate). The number of replicates (*n*) is shown for each graph. Student’s *t*-tests (two-tailed, unpaired) were used to compare features that followed a normal distribution; the Mann–Whitney *U*-test (two-tailed) was used to compare non-parametric features. Data are mean± s.e.m., *P<0.05, **P<0.01, ***P<0.001; ns, not significant.

We established filming methods using a specially designed tank and camera setup that allows consistent video recordings free of reflections and shadows (Fig. 3A). We also wrote a computer program (ShadowFish) to generate a minimal set of data to characterise fish movement; this greatly reduces the dimensionality of the data, which is essential for input into computational analyses. ShadowFish software and tank design are freely available (github.com/ghughesyork/ShadowFish). The position of the fish spine is approximated in each frame of an input video clip, and then the flexion or bending angle is measured at five equidistant positions along the spine (Fig. 3B). Seven *x*,*y* coordinates were also measured along the spine for each frame. Simplifying the video data in this way facilitates our subsequent classification analyses using EAs. Forty-six *dj-1^−/−^* mutant zebrafish (12 weeks old) and the same number of age-matched wild-type controls were recorded swimming for 5 min following an acclimation time of 10 min after moving into the ShadowFish tank. Using *dj-1^−/−^* and wild-type fish from multiple different breedings reduced the chances of evolving a classifier that recognised a pattern occurring in a single progeny. The increased generality of the evolved classifier improves classification accuracy when used to discriminate future *dj-1^−/−^* mutants. Then, 30-s video clips, filmed at 100 frames/s, were processed from recordings. Two 30-s clips from each recording were processed using the ShadowFish software; these were visually assessed to ensure that we removed any video with a reflection or a shadow or where the fish was not in view. The 30-s clips were also processed using the ShadowFish software to collect movement data in the same way from two other genotypes, *pink1*^−/−^ and *dmd*^ta222a/+^.

### Extracting features of movement

Initial analyses were undertaken using MATLAB to calculate selected features of movement including distance travelled (Fig. 3C), velocity (Fig. 3D), time spent moving (Fig. 3E) and swimming episode duration (Fig. 3F). Over a 30-s period, *dj-1^−/−^* zebrafish covered significantly less distance at a slower velocity compared to wild-type fish. While wild-type fish spent 96% of their time moving, *dj-1^−/−^* mutants were moving only 83% of the time. Additionally, the mean swimming episode duration by a *dj-1^−/−^* was 17 s, compared to 25 s for the wild type. Tail beat frequency (Fig. 3G) and mean tail bend amplitude (Fig. 3H) were analysed at low, medium and high speeds of movement (Keatinge et al., 2015). It was hypothesised that mutant fish might need to work harder to achieve higher speeds through more tail beats; however, only the tail beat frequency at low speed was significantly affected and reduced in the *dj-1^−/−^* mutant. A trend was seen in the wild-type zebrafish for the tail bend amplitude to decrease with an increase in swimming speed, suggesting that momentum allows fish to coast once higher speeds have been reached, reducing tail bend amplitude and energy expenditure (McHenry and Lauder, 2005). In contrast, the mean tail bend amplitude is significantly less in the *dj-1^−/−^* zebrafish at low speeds but significantly greater at high speeds, and a greater tail bend amplitude was observed in the Dj-1-deficient zebrafish at high speeds compared to the wild type (Fig. 3H). As the *dj-1^–/–^* mutant fish stop more frequently (Fig. 3F), the resulting inertia might impede them from gaining the momentum required for coasting. Alternatively, the *dj-1^−/−^* zebrafish could lack the fine motor control required to moderate their swimming at speed. This is similar to the hypothesis that PD patients fail to appropriately scale the size of their movements to complete a given task (Mazzoni et al., 2012). In contrast to the differences in movement seen at 12 weeks post-fertilisation (wpf), we found that, in younger zebrafish (8 wpf), most features of movement of *dj-1* mutants were not significantly different from those of age-matched controls (Fig. S1). This progressive impairment of movement in *dj-1^−/−^* mutant fish is consistent with the progressive loss of motor control seen in PD patients (Greffard, et al., 2006).

### Evolving classifiers using extracted features of movement

Machine learning has proved successful in analysing highly complex, non-linear data in human PD patients (Lones et al., 2013), and we have adapted the methods from our previous study to this zebrafish model of PD. Automated analyses of the digitised swimming data were used to measure selected features of movement in the mutant and wild-type fish, with the aim to distinguish any combination of features that characterises the movement of the *dj-1^−/−^* zebrafish. In addition, we included data extracted from another parkinsonian model, the *pink1*^−/−^ mutant zebrafish, and from zebrafish heterozygous for *sapje* (*dmd^ta222a/+^*) (Bassett et al., 2003). *sapje* is a recessive mutation that is lethal when homozygous and a model for muscular dystrophy, another human disease in which movement is disrupted. The *dmd^ta222a^* allele is recessive so in a heterozygote condition there is no expected phenotype or expected change in movement, thus providing a negative control for evolving classifiers.

We describe here an objective method, using machine learning, to identify any movement phenotype that can distinguish mutants from the wild type with a defined level of accuracy. Machine learning can automatically analyse large datasets and ‘learn’ to recognise differences between the classes of data (Alpaydin, 2016). We use a form of ‘supervised learning’, where a supervisor provides the desired output for every input when generating an optimised classifier (Ayodele, 2010). In this instance, the input is all the features of movement extracted from the recording of a fish and the desired output is the class of fish, either mutant or wild type. An EA is a population-based process designed to optimise the solution to a defined problem; by evaluating a population of candidate solutions using a ‘fitness function’ (an objective mathematical measure that determines the most effective solution) and subsequently spawning a new population of candidate solutions for the next generation. The EA goes through multiple generations and the candidate solution becomes increasingly optimised with each round of evolution. This culminates in an effective solution being produced and expressed as a discrete mathematical equation that describes the solution (Smith et al., 2015). Moreover, EAs are one of the few ‘white box’ forms of machine learning for which the functions that make up the classifier can be inspected (Lones et al., 2013); this is a useful characteristic to allow discovery of any movement phenotype characteristic of a zebrafish model of PD.

**Fig. 4.**
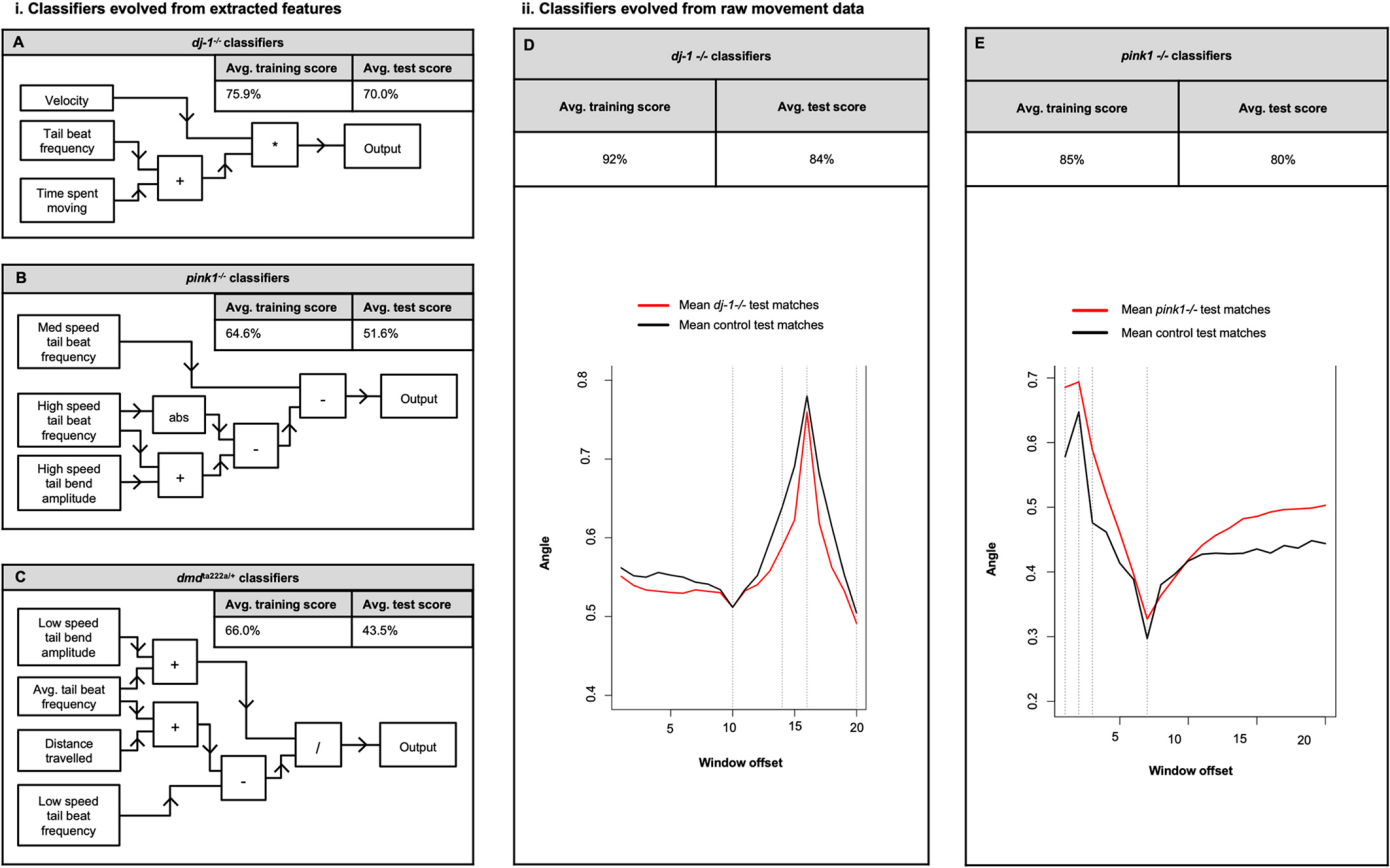
**Machine learning evolves classifiers from movement data and discriminates *dj-1*^−/−^ zebrafish as distinct from controls.** (i)(A-C) An analysis using an evolutionary algorithm to discriminate a classifier by analysing extracted features of movement. (A) The mean training and test scores for classifiers evolved to recognise *dj-1*^−/−^ zebrafish (*n*=30) at 12 wpf over 20 folds of datasets containing extracted features of movement. The CGP network of the highest scoring *dj-1*^−/−^ classifier evolved using the extracted features is depicted as a flow diagram. (B) The mean training and test scores for classifiers evolved to recognise *pink1*^−/−^ zebrafish (*n*=37) at 14 wpf over 20 folds of dataset containing extracted features of movement. The CGP network of the highest-scoring *pink1*^−/−^ classifier evolved using the extracted features is depicted as a flow diagram. (C) The mean training and test scores for classifiers evolved to recognise *dmd^ta222a^*^/+^ zebrafish (*n*=25) at 12 wpf over 20 folds of dataset containing extracted features of movement. The CGP network of the highest-scoring *dmd^ta222a^*^/+^ classifier evolved using the extracted features is depicted as a flow diagram. (ii)(D,E) A separate analysis of the raw movement data using sliding window classifiers trained with the PC2 time series data to generate symbolic mathematical expressions that describe discriminatory local patterns of movement within the data. (D) The training and test accuracies for the classifier evolved to recognise *dj-1*^−/−^ zebrafish at 12 wpf. Mean plots of the PC2 time series data in the 20 windows most useful for discriminating *dj-1*^−/−^ mutants (*n*=36) (red) and age-matched controls (*n*=64) (black) in the test dataset. (E) The training and test accuracies for the classifier evolved to recognise *pink1*^−/−^ zebrafish at 14 wpf. Mean plots of the PC2 time series data in the 20 windows most useful for discriminating *pink1*^−/−^ mutants (*n*=39) (red) and age-matched controls (*n*=44) (black) in the test dataset.

Using selected features of movement, an EA was used to train classifiers labelling fish as either *dj-1^−/−^* or wild type. Forty-six *dj-1^−/−^* and 46 age-matched control zebrafish were recorded swimming for a period of 5 min. Two separate 30-s clips were processed from each 5-min recording, generating 92 comma-separated value (CSV) files containing raw movement data for each class of fish. After visual quality checks, 30 recordings of *dj-1^−/−^* and an equal number from age-matched wild-type controls were used to evolve classifiers. The type of EA used was a Cartesian genetic programming (CGP) algorithm, in which programs are represented as directed graphs with two-dimensional grids of computational nodes (Miller, 2011). CGP was selected as it is a white box machine learning algorithm that is suitable for relatively small sample sizes and can provide insight into the contribution of respective features in the resulting classifier, as demonstrated in previous work in analysing human movement disorders (Lones et al., 2013). Here, we use 30 recordings, and 60 data points were obtained for each class, which, according to the methods of Bland and Altman (2007), provides a 95% confidence interval of ±0.44 standard deviations. The CGP network for classifying *dj-1^−^*^/−^ fish that achieved the highest test score on a fold of datasets is shown in Fig. 4A. Velocity, mean tail beat frequency and time spent moving were the extracted features of movement used in the highest-scoring *dj-1^−^*^/−^ classifier. These data reveal these features of movement as the most useful for discriminating the *dj-1^−/−^* mutants from wild type. The mean test score of classifiers evolved using the 20 folds of datasets was 70%, indicating that an equation could distinguish *dj-1^−^*^/−^ fish by their features of movement 70% of the time. Similar analyses were carried out to classify extracted features of movement from *pink1*^−/−^ (another PD model) and *dmd^ta222a^*^/+^ (a negative control) and are presented in Fig. 4B,C. The EA was unable to compute an equation to distinguish these genotypes from controls using extracted features of movement (test scores were 51.6% and 43.5%, respectively).

### Evolving sliding window classifiers from raw movement data

For an unbiased classification we used the raw data extracted from the videos, without selection of any particular features of movement, for input into an EA. To do this, we processed the movement data using principal component analysis (PCA) to condense the data into principal components (PCs) by linear combination of the tail bend angles in each frame (Fig. S2). This reduced the dimensionality of the tail bend angles, transforming the multivariate time series into a univariate time series, while retaining the inherent variation of the data. PC2 was used to evolve classifiers as it retained the high frequency movement and subtle variation important for characterising a movement phenotype (Lones et al., 2013).

Sliding window classifiers, encoded using CGP, were evolved to classify the mutants using the PC2 time series as input data. The mathematical equations generated describe local patterns in the movement data that can be used for discrimination (Lones et al., 2013). The window in a sliding window classifier contains the data from a specific range in a time series, which become the input data for the evolved algorithm. The raw movement data extracted from 36 clips of *dj-1^−^*^/−^ mutants and 64 clips of age-matched control zebrafish with visually assessed, high-quality tracking were used to evolve sliding window classifiers. Here, 20 PC2 values (extracted from 20 frames of a recording) were used as the input data for a window. The algorithm applies pre-defined functions to the input data and produces a continuous value between 0 and 1 (Lones et al., 2013). Subsequently, the window slides along one position in the time series to get the next overlapping range of data points. The algorithm is applied to the new input data and produces a further output value; the process is repeated until the sliding window has reached the end of the time series. The final classifier output (the mean of all the output values) labels the data as belonging to one of two classes depending on a threshold value. Similar analyses were carried out on the *pink1*^−/−^ homozygous mutants and *dmd^ta222a^*^/+^ heterozygote zebrafish.

One of the aims of this work was to confidently classify zebrafish using a protocol that is effective and practical, by acquiring short 5-min videos for analysis. Two 30-s clips were determined empirically to provide sufficient data to identify differences in movement that allow classification of both the *dj-1^−/−^* and *pink1^−/−^* mutants from control fish. For the *dj-1^−^*^/−^ and *pink1*^−/−^ datasets, the sliding window classifiers were able to discriminate mutant fish around 80% of the time (Fig. 4D,E). The classifiers evolved using the raw movement data were more effective than classifiers evolved using select features. This is likely due to the objectivity that is possible using a continuous stream of data as well as the increased amount of data analysed from each recording. In addition, each selected feature was calculated to a single value summarising the movement in a video clip and reducing the data available to the EA. To visualise local patterns in the movement data characteristic of each class of fish [mutant (red) or wild type (black)] a mean plot of the 20 windows that were most useful for discriminating mutants is shown for *dj-1^−^*^/−^ (Fig. 4D) and *pink1*^−/−^ (Fig. 4E) mutant zebrafish. This same set of computational analyses was undertaken using heterozygous *dmd^ta222a^*^/+^ zebrafish and a useful classifier could not be evolved. Fig. S3 reports the training and test areas under the curves (AUCs) for 20 runs, showing a mean of 0.78 for train and 0.43 for test. This shows that classifiers are evolved that over-fit the training data, but these models cannot generalise or classify any meaningful differences between the movement of *dmd^ta222a^*^/+^ and the wild-type zebrafish. Together, these data indicate that machine learning can discriminate movement phenotypes when present, as is the case with the zebrafish models of PD, but not in the heterozygous carrier of the muscular dystrophy gene *dmd^ta222a^*^/+^ that has no phenotype; this was expected given the recessive inheritance of the disease. One limitation of our study is that we did not treat adult animals with drugs; for instance, MPTP treatment mimics PD, which would be a useful addition to our protocols. Moreover, the ability of artificial intelligence to identify PD fish presents the possibility (indeed the next step) of treating the fish with known or potential therapeutics and then re-assessing the same fish. We are currently working with collaborators to develop these further protocols.

### Molecular signatures consistent with parkinsonian pathology characterise *dj-1*^−/−^ brains

**Fig. 5.**
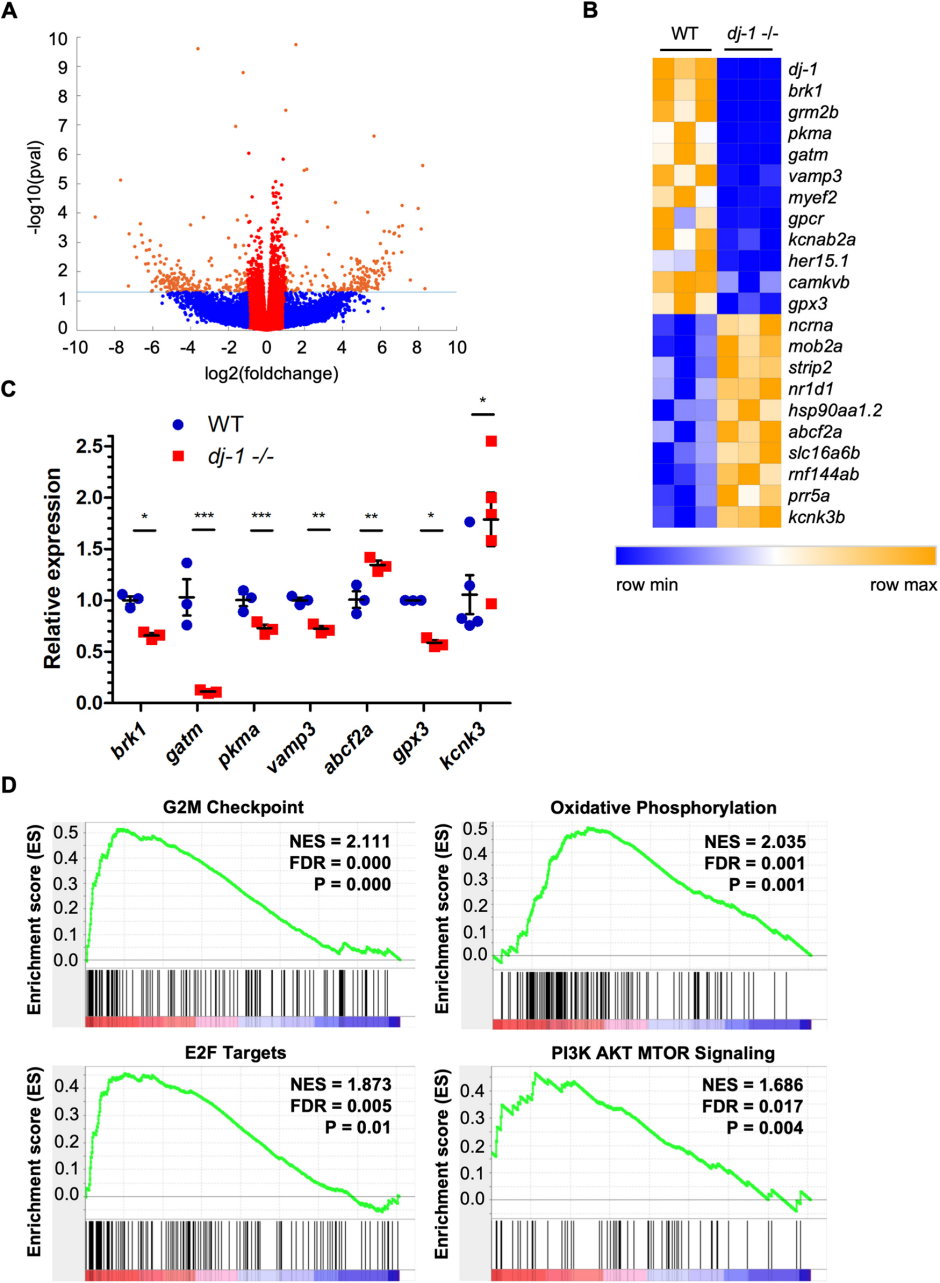
**Differential gene expression in the** ***dj-1*** **^−/−^ mutant brain.** (A) A volcano plot representing the global changes in gene expression observed by RNA-seq analysis, comparing RNA from the brains of *dj-1*^−/−^ mutants (*n*=3, biological replicates) and their wild-type siblings (*n*=3) at 16 wpf. The fold change (log2) of each transcript was plotted against the *P*-value (−log10). Transcripts in red are down/upregulated less than 2-fold. Transcripts in orange that appear above the dotted line are differentially expressed with a *P*-value of <0.05 and down/upregulated more than 2-fold. Transcripts in blue have a *P*-value of ≥0.05. (B) A heatmap showing select genes differentially expressed with an FDR q-value of <0.05. Relative expression is represented on a colour scale from blue (low) to orange (high). (C) qRT-PCR analysis validating the results seen in the RNA-seq using RNA extracted from the brains of *dj-1*^−/−^ and wild-type siblings at 16 wpf (*n*=3 for all but *n*=5 for *kcnk* analysis, biological replicates). Student's *t*-tests (two-tailed, unpaired) were used to compare the dCt values for *dj-1*^−/−^ and wild-type samples. Data are mean±s.e.m., *P<0.05, **P<0.01, ***P<0.001. (D) GSEA enrichment plots for Hallmark gene sets: G2M checkpoint, oxidative phosphorylation, E2F transcription factors and PI3K/AKT/mTOR signalling. FDR, false discovery rate-adjusted *P*-value; NES, normalised enrichment score; P, nominal *P*-value. Data have been deposited in GEO, accession number GSE135271.

Animal models can provide important insight into the molecular basis of genetic disease. To investigate any global changes in gene expression associated with a loss of Dj-1, RNA-seq was carried out on RNA extracted from adult zebrafish brains using *dj-1^−/−^* mutants and three wild-type siblings at 16 wpf. An overview of these effects is shown as a volcano plot (Fig. 5A) and a short list of affected genes was curated using the criteria of fold change ≥1.2 (up- or downregulated) and false discovery rate (FDR)-adjusted *P*-value ≤0.05. The list is depicted as a heatmap illustrating the relative expression of 22 transcripts that were found to have significantly altered expression in the *dj-1^−/−^* mutant brains (Fig. 5B). Validation of a set of these targets by qRT-PCR analysis confirmed the changes in expression for some genes of interest (Fig. 5C). These data have been deposited in NCBI's Gene Expression Omnibus (GEO) (Edgar et al., 2002), under accession number GSE135271 (https://www.ncbi.nlm.nih.gov/geo/query/acc.cgi?acc=GSE135271). A list of all differential transcripts with q<0.05 is provided in Table S2A,B.

Genes significantly affected by loss of DJ-1 include those with cell functions known to be disrupted in PD. *VAMP3*, part of a SNARE complex, found on the vesicle membrane and required for vesicular transport from early, recycling endosomes to the trans Golgi network was found downregulated in HeLa cells (Mallard et al., 2001). DA neurons in the substantia nigra pars compacta (SNc) have increased vulnerability to dysfunctional intracellular trafficking, as vesicles transport cargo great lengths and to a high number of synapses due to their extensive axonal arborisation (Bolam and Pissadaki, 2012; Giguère et al., 2019). Mitochondrial dysfunction is accepted as central to PD pathophysiology (Hu and Wang, 2016). Here, we find that *glycine amidinotransferase* (*gatm*), encoding a mitochondrial enzyme involved in creatine synthesis (Sandell et al., 2003), is significantly downregulated in *dj-1^−/−^* brains. As well as an energy store, creatine has an antioxidative function, scavenging for reactive oxygen species (ROS) (Allen, 2012; Gabriel et al., 2013). In addition, *glutathione peroxidase 3* (*gpx3*), encoding an enzyme that reduces hydrogen peroxide, thereby protecting the cell from oxidative stress, also has reduced expression in the brains of *dj-1^−/−^* zebrafish. Interestingly, the human *GPX3* gene contains a peroxisome proliferator response element (PPRE) that is activated by PPARγ (Chung et al., 2009), a signalling pathway found activated downstream of DJ-1 (Han et al., 2018). *pyruvate kinase M1/2a* (*pkma*), a key regulator of aerobic glycolysis (Sun et al., 2011), is also downregulated, suggesting dysregulated metabolism in *dj-1^−/−^* brains.

In order to take a broader view of the classes of genes sensitive to loss of DJ-1, we used gene set enrichment analysis (GSEA) to analyse our RNA-seq data (Subramanian et al., 2007, 2005) and found enrichment profiles consistent with disrupted metabolism and cell cycle regulation. This method uses pre-defined sets of genes that have been categorised based on interactions in a common pathway or a related biological function. The data from the RNA-seq analysis were processed and a normalised enrichment score (NES) calculated to determine whether genes affected are over-represented in any molecular signature database. The NES reflects the degree to which the genes in a gene set are over-represented at the top or bottom of a gene list ordered by association with a genotype. Our results revealed that *dj-1^−/−^* brains have over-representation of the gene sets presented in Table S3. As would be predicted from several previous reports (Kim et al., 2005; Lan et al., 2017), we find enrichment of gene sets associated with PI3K/AKT/mTOR and mTORC1 signalling (Fig. 5D; Table S3). This is consistent with increased levels of mTOR in mouse models of PD (Wills et al., 2012) and the finding that DJ-1 negatively regulates the tumour suppressor PTEN, which is part of the PI3K/AKT signal transduction pathway (Kim et al., 2005).

Our GSEA also indicates an upregulation of genes associated with oxidative phosphorylation in the absence of Dj-1. Metabolic regulation in neurons is crucial given the high and dynamic energy demands of the vertebrate brain (Yellen, 2018); moreover, SNc DA neurons in particular have high metabolic needs (Giguère et al., 2019). Our results show that some genes coding for players in glycolysis (Fig. 5C) are downregulated, while the gene set for oxidative phosphorylation is over-represented (Fig. 5D; Table S3). We also find the enrichment of the G2 to M transition gene set and targets of E2F transcription factors (Fig. 5D), as well as other cell cycle-related genes sets (Table S3) in the brains of *dj-1*^−/−^ zebrafish. Interestingly, entering the cell cycle has been linked to cell death in neurons (Herrup et al., 2004; Herrup and Yang, 2007), and, moreover, Höglinger et al. (2007) found that the pRb/E2F pathway caused neuronal cell death in a chemical model of PD, consistent with the over-representation of mitotic gene sets in *dj-1^−/−^* brains, reflecting their neurodegenerative pathology.

## DISCUSSION

Age-dependent neurodegeneration is difficult to model in organisms with short lifespans, and, moreover, motor disorders are not always apparent and can be difficult to measure (Lee et al., 2017; Rousseaux et al., 2012). There has been some success modelling PD using neurotoxin-based models in the mouse; however, these present a rapid loss of DA neurons, which does not align with the progressive neurodegeneration of PD in humans (Jackson-Lewis and Przedborski, 2007). Genetic approaches for modelling PD in mice have had less success in reproducing the neurodegeneration. The loss of *Pink1*, *Prkn*, *Dj-1* or *Fbxo7* in the mouse does not reduce the number of nigrostriatal DA neurons or locomotor ability (Gispert et al., 2009; Kim et al., 2005; Perez and Palmiter, 2005; Vingill et al., 2016). A further triple knockout of *Pink1*, *Prkn* and *Dj-1* in the mouse still failed to produce a phenotype with recognisable aspects of PD (Kitada et al., 2009). In contrast, transgenic expression of genes associated with autosomal dominant PD in humans, *LRRK2* (G2019S) and *SNCA*, was shown to cause a loss of nigrostriatal DA neurons and locomotor defects in the mouse (Ramonet et al., 2011; Janezic et al., 2013). In addition, aggregates of α-synuclein forming in the motor neurons of transgenic mice overexpressing α-synuclein has been reported, which could impact the movement phenotype (van der Putten et al., 2000). Rats may prove a better mammalian model for PD; Dave et al. (2014) reported a loss of DA neurons and locomotor defects in rats lacking *Dj-1* and *Pink1*, but not *Prkn*. Overexpression of LRRK2 (G2019S) and SNCA in the rat brain has been able to cause a loss of the nigrostriatal DA neurons in some cases (Dusonchet et al., 2011; Lo Bianco et al., 2002). Nonetheless, compared to a mouse or rat models, there are advantages to using zebrafish in drug screens. Zebrafish are smaller, have lower maintenance costs and produce much larger broods for generating large numbers of animals for analyses; there is also the simplicity of administering candidate drugs in their water. Although the method described here uses adults and is not suitable for the high-throughput drug screens used on zebrafish larvae (Gallardo et al., 2015), it may provide a valuable addition to current drug screening protocols targeted at genetic diseases that have no larval phenotype but present a later movement disorder, as part of a multifaceted approach (Volpatti et al., 2020).

### An unbiased method to discriminate movement disorders in zebrafish

Here, we have used an EA to classify data obtained from recordings of zebrafish movement by measuring the bending of the spine. Previous tracking software has analysed location coordinates as fish swim; this is done in larvae and adults and informs only on the location or locomotor activity of individuals or groups of fish (Stewart et al., 2015; Cronin and Grealy, 2017). In contrast, we have measured the tail bend angle along the back of individual adults, to inform on muscle movement and assess dyskinesia. Using this approach, machine learning discriminates a parkinsonian phenotype in zebrafish deficient in *dj-1/park7*, a gene that causes a recessive form of early onset PD in humans. Off-target effects are a worry of any experimental program that relies on gene targeting. Back crossing to the wild-type strain for several generations can remove any potential modifiers linked to the targeted gene, and extending our study in this way would ensure that the movement phenotypes distinguished in our analyses tracked with the mutant allele. It is also important that the fish are genetically similar, except for the allele of interest, making it difficult to compare across different generations; therefore, the fish compared were always cousins of the same generation. In addition, raising fish in separate tanks and the emergence of traits from unrelated homozygous mutations resulting from in-crossing wild-type fish are potential caveats to our interpretations. Nonetheless, machine learning was able to distinguish *dj1^−/−^* and *pink1^−/−^* mutants from wild-type cousins, but could not evolve an equation to discriminate between our control line, *dmd^ta222a/+^* heterozygotes, from wild-type fish, which were also raised in separate tanks.

Machine learning has also been used to classify mouse models of PD generated by injection of neurotoxins directly into the brain; the pronounced movement phenotype is measured using the well-established Noldus Catwalk gait analysis and discriminated with an accuracy of 96% (Frohlich et al., 2018). Similarly, the extraction of movement data and evolution of classifiers to diagnose PD in humans has been refined to reach accuracies of up to 95% (Lones et al., 2013). Our novel ShadowFish tracking software extracts data suitable for analysis by EAs and results in classifiers with ∼80% accuracy. The ability of our new method to evolve algorithms that discriminate movement of zebrafish carrying two different PD, but not that of zebrafish carrying a heterozygous mutation in *dmd*, indicates that our method has the potential to reach higher classification accuracies and provide a valuable new method to detect movement disorders in a non-mammalian vertebrate model.

Clinical diagnosis of PD is challenging, with an accuracy of ∼80% (Hughes et al., 1992; Rizzo et al., 2016). More accurate, less subjective diagnosis of PD using EAs has improved diagnostics and is able to describe the bradykinesia characteristic of PD in a finger-tapping exercise (Lones et al., 2013), allowing early diagnosis of PD distinct from other neurodegenerative diseases. Our adaptation of this technology to evolve classifiers that identify mutant zebrafish based on movement data will provide a method to screen for drugs that improve the movement phenotype. We have found that Dj-1-deficient zebrafish display an overall loss of movement measured as a reduction in distance travelled, velocity, time spent moving and duration of a swimming episode; comparable to the bradykinesia observed in PD patients (Jankovic, 2008). Furthermore, the shorter duration of swimming episodes indicated more frequent periods of inactivity, akin to the freezing episodes observed in PD (Chee et al., 2009). There is, however, a great deal of subjectivity when studying features selected based on conventional movement measures. In comparison, the sliding window classifier is much more objective, automatically extracting features based on minute differences in movement that are undiscernible to the human eye, rather than the general features of movement. On examination of the input video data, we find that the discriminating classifiers often use data from when the fish turns. This suggests that the movement phenotype in parkinsonian zebrafish is similar to that found in PD patients, in whom bradykinesia is particularly evident when turning around (e.g. Chou and Lee, 2013). A further consideration to take into account is that a muscle disorder might be contributing to the movement phenotype, as loss of *dj-1* has been found to affect metabolic respiration in skeletal muscle cells and result in overall reduced body mass (Edson et al., 2019).

### Molecular signature of a PD brain: increased oxidative phosphorylation and cell cycle re-entry

Confirmation of a valid parkinsonian model was provided by GSEA (Subramanian et al., 2005), which revealed molecular signatures described in other genetic models of PD. The DJ-1 protein is sensitive to ROS (Canet-Avilés et al., 2004; Mitsumoto et al., 2001), acting as a neuroprotector by quenching excess ROS at the mitochondrial membrane (Taira et al., 2004) and is also required for the subcellular localisation of hexokinase (HK1) to the mitochondria (Hauser et al., 2017). This links DJ-1 activity directly to mitochondrial function, alongside other notable autosomal recessive PD genes *PINK1* and *PRKN* that monitor and maintain mitochondrial function. Mitochondrial dysfunction is accepted to be a major factor in the pathology of PD (reviewed in Cookson, 2012), although it is puzzling how defective mitochondria, organelles that are essential in all cells, leads to the specific depletion of SNc DA neurons. One explanation is that the high number of synapses associated with these neurons results in extraordinarily high energy demands (reviewed in Bolam and Pissadaki, 2012); indeed, there is a higher basal rate of mitochondrial oxidative phosphorylation in SNc DAs compared to DA neurons of the ventral tegmental area (Pacelli et al., 2015). Recently, good evidence for this hypothesis has been provided by the Trudeau laboratory, when they showed that a mouse engineered with increased axonal arborisation of SNc DA neurons is more vulnerable to PD-causative neurotoxins (Giguère et al., 2019). Our findings that genes associated with oxidative phosphorylation are altered in the *dj-1*^−/−^ zebrafish is consistent with the notion that disruption of bioenergetics is a key feature of a neurodegenerative brain.

Upregulation of genes associated with oxidative phosphorylation in a mouse *Prkn*^−/−^ model (Giguère et al., 2018) and a zebrafish *dj-1^−/−^* model of PD has been described (Edson et al., 2019), while dysregulation of genes associated with metabolism is consistent with a neurodegenerative state in PD (Giguère et al., 2018; Shi et al., 2015) as well as Alzheimer's disease (Demetrius et al., 2015). One interpretation (Yellen, 2018) is that the high energy demands in neurons requires the uncoupling of glycolysis and oxidative phosphorylation, and it may be that DJ-1 is required for this switch. Another interpretation is that neurodegeneration results from an ‘inverse Warburg effect’; this has been proposed as a cause of sporadic Alzheimer's disease, in which oxidative phosphorylation is upregulated in ageing or otherwise impaired neurons as a compensatory response to meet energy needs (Demetrius et al., 2015), an idea supported by our data that oxidative phosphorylation genes are enriched in the absence of Dj-1.

GSEA also reveals that in zebrafish brains lacking Dj-1, there is an enrichment of genes associated with mitosis, such as those regulating the G2/M checkpoint, E2F target genes, as well as the cancer-related gene sets associated with epithelial mesenchymal transition, Myc targets and the mitotic spindles. This might seem surprising, because DJ-1 was first identified by its interaction with Myc and indeed can transform 3T3 cells when co-expressed with Myc or Ras (Nagakubo et al., 1997). However, PD genes have been implicated in cell cycle control (reviewed in West et al., 2005) and the re-activation of cell cycle proteins (including the E2F factors) has been detected in tissue from PD patients (Höglinger et al., 2007). Post-mitotic neurons have mechanisms in place that prevent cell cycle progression, and re-entering the cell cycle can lead to apoptosis; this may be a mechanism underlying neurodegeneration (reviewed in Folch et al., 2012; Herrup et al., 2004; Herrup and Yang, 2007). We conclude that the over-representation of mitotic gene sets, together with those indicative of altered metabolism, reflects the neurodegenerative state of *dj-1^−/−^* zebrafish brains.

### Machine learning can contribute to drug development

To date, most zebrafish models of PD have been transient models in larvae, generated using neurotoxins such as MPTP, which cause a rapid loss of the DA neurons (Bretaud et al., 2004; Feng et al., 2014), or by morpholino knockdown of PD-associated genes (Bretaud et al., 2007; Flinn et al., 2009; Zhao et al., 2011); in addition, an adult *pink1*^−/−^ mutant zebrafish has been described (Flinn et al., 2013). A few studies have tested potential therapies on zebrafish models of PD by measuring the distance travelled and velocity of larvae (Cronin and Grealy, 2017; Sheng et al., 2010). PD in humans is both age related in nature and characterised by a movement phenotype of bradykinesia (slowing of movement), resting tremor and muscle rigidity (Jankovic, 2008). Given the progressive loss of DA neurons, an adult model of PD is intuitively more suitable, and evolving classifiers using tail bend data provides a more comprehensive, unbiased assessment of the movement phenotype. The novel use of machine learning described here has the potential to accelerate the drug discovery pipeline (Lam and Peterson, 2019) by allowing more comprehensive screening using non-mammalian vertebrate models of movement disorders.

## MATERIALS AND METHODS

### gRNA design

A synthetic gRNA was designed to target Cas9 cleavage in *dj-1* upstream of the codon for residue C106, in order to produce an early stop codon and loss of C106 from the translated protein. This cysteine is critical for the oxidative stress response of DJ-1 (Wilson, 2011). The online CRISPR design tool ChopChop (https://chopchop.cbu.uib.no) was used to identify a target sequence near the start of the *dj-1* coding sequence. A 20 bp target sequence (5′-GCCGGTTCAGTGCAGCCGTG-3′) in exon 2 of *dj-1* was selected and incorporated into the forward primer for gRNA production. A promoter sequence for increasing transcription efficiency with T7 RNA polymerase was added to the 5′ end of the forward primer along with an additional G nucleotide necessary for the T7 polymerase to work (Nakayama et al., 2014). This resulted in the following forward primer sequence: 5′-GCAGCTAATACGACTCACTATAGGCCGGTTCAGTGCAGCCGTGGTTTTAGAGCTAGAAATAGCAAG-3′. *pink1* was disrupted using the same approach, with the modification of two gRNAs designed 100 bp apart: *pink1*(1) 5′-GCAGCTAATACGACTCACTATAGGGTGAAGCAGAAAGTCGAAGGTTTTAGAGCTAGAAATAGCAAG-3′ and *pink1*(2) 5′-GCAGCTAATACGACTCACTATAGCGCAGCTGTTTATGAGGCTGGTTTTAGAGCTAGAAATAGCAAG-3′. The reverse primer used for gRNA synthesis is common to all gRNAs: 5′-AAAAGCACCGACTCGGTGCCACTTTTTCAAGTTGATAACGGACTAGCCTTATTTTAACTTGCTATTTCTAGCTCTAAAAC-3′ (Nakayama et al., 2014). For genotyping, the primers to amplify the *dj-1* genomic region were as follows: For, 5′-GGCTCTGGCCATCATTACTAT-3′; Rev, 5′-GTAAAGTCAGACCTGTTTGTGTG-3′. Primers to amplify the *pink1* genomic region were as follows: For, 5′-GGCTGTATTTAGAAAGAAGAAGTTTCAG-3′; Rev, 5′-GCAGCACAGTACAATTGTCAACTATAAA-3′.

### Generating mutant lines

The strain of zebrafish used was London Wild Type (LWT) and both males and females between 8 wpf and 14 wpf were analysed for movement phenotypes. Transcriptomic analyses were carried out on the brains of zebrafish at 16 wpf and the immunohistochemical analysis was carried out on the brains of zebrafish at 12 mpf. This study was carried out using procedures authorised by the UK Home Office in accordance with the Animals Scientific Procedures Act (1986) and approved by the Animal Welfare and Ethical Review Body at the University of York and the UK Home Office.

Cas9 protein was co-injected with *dj-1*-targeting single-guide RNA into single-cell embryos from the LWT zebrafish strain to produce the F0 fish that were outcrossed with wild type to generate heterozygous F1 mutants. Genotyping of the F1s revealed a male and female carrying the same mutation in *dj-1*; these heterozygotes were in-crossed to produce the F2 generation. F2 zebrafish homozygous for *dj-1* were out-crossed with wild type to generate a stock of heterozygous fish (F3); these were in-crossed and the offspring raised together (F4), genotyped, and used for molecular analysis and generating stocks of homozygous and wild-type fish for movement analysis. Genotyping the *dj-1* locus in zebrafish was carried out by PCR of the target region followed by BbvI restriction digest assay; digestion of wild-type *dj-1* amplicon results in products of 214 bp and 55 bp in length. The BbvI restriction site is lost in the mutated *dj-1* so the PCR product remains 286 bp in length. *pink1* was disrupted using two gRNAs designed 100 bp apart, resulting in a 101 bp deletion detectable by gel electrophoresis following PCR of the target region. The breeding strategy was the same as described above.

### Western blotting

Zebrafish brains were dissected from adults and flash frozen in liquid nitrogen before storing at −80**°**C. Frozen brains were homogenised in 100 μl lysis buffer consisting of 50 mM Tris-HCl (pH 7.4), 10 mM sodium glycerophosphate, 10 mM sodium pyrophosphate, 150 mM NaCl, 5 mM MgCl_2_, 1 mM EDTA, 1 mM DTT, 1% Triton X-100, 10% glycerol, 1 mM sodium orthovanadate and cOmplete™Mini EDTA-free Protease Inhibitor Cocktail (Roche). The lysate was centrifuged at 21,000 ***g*** for 10 min prior to running on a 12% SDS-PAGE gel. After transfer to a PVDF membrane, immunodetection used the anti-Dj-1 polyclonal antibody (PA5-72638, Invitrogen) and anti-Gapdh monoclonal antibody (G8795, Sigma-Aldrich), both at a dilution of 1:50,000. Horseradish peroxidase (HRP)-linked anti-rabbit IgG (7074, Cell Signaling Technology) and HRP-linked anti-mouse IgG (62-650, Invitrogen) were used at concentrations of 1:2000 and 1:4000, respectively.

### qRT-PCR

Zebrafish brains were dissected out of adults before homogenising in 1 ml TRIzol reagent (Sigma-Aldrich) for RNA extraction. Following centrifugation at 4°C for 10 min at 21,000 ***g***, RNA was purified from the upper aqueous phase using the RNA Clean & Concentrator Kit (ZYMO) following the manufacturer's instructions. Complementary DNA (cDNA) was synthesised from 1 μg RNA by reverse transcription using Superscript IV Reverse Transcriptase (Thermo Fisher Scientific) following the manufacturer's instructions with random hexamer primers. Primers were designed to amplify a product of 50-150 bp with one primer in each pair crossing an exon junction (Table S1). Each quantitative PCR analysis was carried out with a minimum of three biological replicates and three technical repeats. The mean cycle threshold (Ct) value was normalised to the Ct of reference gene *ef1ɑ* (also known as *eef1a1l1*), generating a delta-Ct (dCt) value for genes of interest. Delta-delta-Ct (ddCt) values were then calculated by subtracting the dCt values of wild-type siblings from mutants and the relative fold change was equal to 2^(−ddCt)^. Student’s *t*-tests were carried out comparing the dCt values of mutants and wild-type siblings for each gene. GraphPad Prism5 was used to generate graphs showing the relative fold change with error bars representing s.e.m.

### Immunofluorescence

Adult zebrafish were euthanised at 12 mpf, and whole brains were extracted and fixed overnight at 4°C in a 4% paraformaldehyde/PBS solution. Brains were washed twice in PBS for 10 min. Brains were then sectioned transversely in 3% agarose/PBS by vibratome at a thickness of 100 μM. Based on the protocol by Matsui and Sugie (2017), brains were incubated in 10 mM sodium citrate buffer (pH 8.5) for 2 h at 80°C. Sections were washed twice in PBS with 1% Triton X-100 (PBS/1% Tx) for 15 min. Sections were then blocked in 2% bovine serum albumin (BSA)/PBS/1% Tx for 30 min. After blocking, sections were incubated overnight at 4°C with a 1:500 dilution of mouse anti-Th (22941, Immunostar) in 2% BSA/PBS/1% Tx. Sections were then washed four times in PBS/1% Tx for 30 min. Sections were incubated overnight at 4°C with a 1:500 dilution of goat anti-mouse IgG1 conjugated to Alexa Fluor 488 (A21121, Invitrogen) in 2% BSA/PBS/1% Tx. Sections were then washed four times in PBS/1% Tx for 30 min. Staining for nuclei was carried out by incubating the sections in 1 μg/ml Hoechst 33342 in PBS/1% Tx for 10 min. Sections were then washed four times in PBS/1% Tx for 30 min. Sections were mounted on 15 mm cavity microscope slides before imaging.

### RNA-seq

RNA was extracted and purified as described above from the brains of three *dj-1^−/−^* mutants and three wild-type siblings at 16 wpf. A NanoDrop ND-1000 Spectrophotometer was used to quantify each RNA sample and the RNA integrity was measured using an Agilent 2100 Bioanalyzer. The NEBNext^®^ Poly(A) mRNA Magnetic Isolation Module was used to isolate mRNAs from the total RNA before preparing cDNA libraries using the NEBNext^®^ Ultra RNA Library Prep Kit for Illumina^®^ following the manufacturer's instructions. Unique adaptor sequences were added to the fragments in each cDNA library before pooling them together. The pooled cDNA libraries were then sequenced using 2×150 bp paired end reads on one lane of an Illumina^®^ HiSeq 3000 system. Cutadapt 1.16 was used to trim the adaptor sequences from each read. The fastq files generated were then aligned to the genome assembly GRCz11, the transcriptome was annotated using RefSeq (NCBI) and transcript abundance was quantified using Salmon 0.10.2. Differential expression analysis was then carried out on the aligned transcripts using Sleuth 0.30.0. The likelihood ratio test was used to calculate statistical significance and the Wald test was used to calculate the beta values of transcripts, analogous to fold change. A volcano plot of −log10(pval) against log2(fold change) was generated using MATLAB, and a heatmap representing the changes in expression of a curated set of these transcripts was created by uploading the transcripts per million (TPM) values to https://software.broadinstitute.org/morpheus/.

### GSEA

A GSEA was performed using the GSEA 4.0.0 (Broad Institute) software on the RNA-seq data. All of the genes from the RNA-seq data were used in the GSEA to identify gene sets enriched in a certain phenotypic class. Permutations of the phenotype or genotype labels are used to calculate the statistical significance of a gene set enrichment score (Subramanian et al., 2005). Following instructions on the Broad Institute website (software.broadinstitute.org/gsea) an expression dataset file (.gct) was created containing the TPM values for each transcript, and a phenotype labels file (.cls) was created to label the phenotype of each sample. The Zebrafish.chip file was used to translate zebrafish transcript names to Human Genome Organisation (HUGO) gene symbols. GSEA was then performed to detect enriched HALLMARK gene sets from the Molecular Signature Database (Broad Institute). Gene set permutation was used to assess statistical significance of the enrichment scores as the number of samples in a phenotype was less than 7 (software.broadinstitute.org/gsea). Gene sets with a normalised enrichment score (NES)≥1.5, FDR q-value ≤0.05 and nominal *P*-value ≤0.05 were considered statistically significant.

### Video capture

An Aquatics Habitat breeding tank was modified to record zebrafish swimming while keeping the fish in frame and eliminating any reflection and shadow. To achieve this, a white plastic insert of 145 mm×90 mm was designed to fit the bottom of the tank, and a hollow square frustum with openings at the top and bottom was created with a camera fitted above (Fig. 3A). The insert allowed for background subtraction and the frustum kept the fish in frame and reduced reflections and shadows. A raised platform on the lid positions the camera so that the fish remain in frame at all times**.** Following an acclimation time of 10 min, zebrafish were recorded for 5 min. Recordings were carried out between the hours of 14:00 and 17:00 using a GoPro Hero 3 (GoPro, San Mateo, CA, USA) at 100 frames/s at a resolution of 1280×720 pixels.

### Extracting data

The computational processing resources required to analyse a full 5-min recording are prohibitive, and previous work on assessing movement disorders in humans found that 30-s samples were sufficient (Lones et al., 2013). Consequently, two 30-s clips were selected for processing from the first and second halves of the 5-min recording using bespoke ShadowFish tracking software. Inspired by methods used for larval fish (Budick and O'Malley, 2000), this software was written to identify a fish using background subtraction and trace its midline, dividing it into six parts of equal length (Fig. 3B). For each frame of recording, the absolute *x* and *y* coordinates of the two end points and five vertices along the spine are extracted, in addition to the angle at each vertex. This produces five bend angles and seven *x*,*y* coordinates from each frame. ShadowFish automatically determines the lateral orientation of fish by the direction of movement and therefore the normalised bending angle can be determined accordingly. Each processed video clip was visually assessed before being used to calculate the features of movement or evolve a classifier. Any clip in which the fish was out of the field of view, or the ShadowFish tracking software misidentified a reflection or shadow of the zebrafish for the fish itself, was removed. The extracted movement data are written to a CSV file.

### Calculating features of movement

MATLAB (MathWorks, Natick, MA, USA) was used to calculate features of movement, inspired by previous studies (Table S4), from the extracted raw movement data. The distance travelled by a zebrafish in a recording was determined by taking the absolute coordinates of the fish every 100 frames and using the Pythagoras theorem to work out the distance covered in between. It was necessary to establish when a fish was stationary so data points at those times were not included in certain calculations of features. When a fish travelled less than 5 mm over a 1-s period it was classed as stationary for that second. The time spent moving was the number of seconds in a clip that the fish travelled over 5 mm out of the total 30 s, displayed as a percentage. The mean velocity was calculated by dividing the distance travelled by the number of seconds spent moving. Swimming episode duration was how long a fish was classed as moving before a stationary period. To study the tail beat frequency of a zebrafish in a recording, the sum of the five angles along the spine was plotted over time. A five-frame moving average was also used to help reduce any noise in the angles from the raw data. The points of maximum tail bend were identified as the peaks on the plot. Only peaks with a minimum prominence of five degrees and a minimum separation of five frames were identified, to leave out spikes in the data that were unlikely to be full tail bends. Tail bend amplitudes were the angles identified at the peaks of the plot. Tail beat frequencies and tail bend amplitudes were also calculated at different speeds, in case a difference in movement phenotype was more apparent at a certain speed. Low-speed movement was defined as 0.5≤X<2 cm/s. Medium-speed movement was defined as 2≤X<4 cm/s and high-speed movement was ≥4 cm/s.

### Methodology and statistics of movement data analyses

Multiple features of movement were extracted from the mutant and wild-type fish and all parameters that were measured have been reported. In order to raise and film the numbers of fish required for movement analyses, two or more in-crossings of F4 homozygous mutants or homozygous wild-type siblings were carried out. Statistical analysis of the comparison of movement in wild-type and mutant fish produced the *P*-values quoted, derived from either a Student's *t*-test, if the data were found to be normally distributed, or a Mann–Whitney *U*-test, if the data were non-parametric. Statistical analyses were carried out using GraphPad Prism5 to assess differences in the features of movement for mutant zebrafish and age-matched controls. Any features extracted that contained no numerical value were removed prior to analysis. For example, if a fish only travelled at low and medium speed in a clip then the high-speed tail beat frequency extracted would contain no numerical value and would therefore be left out of future analyses. The features containing numerical values were then examined using the Shapiro-Wilk test to see whether they followed a normal distribution. If the data followed a normal distribution, the interquartile range (IQR) was used to identify suspected outliers. Any values at least 1.5×IQR below the lower quartile or greater than the upper quartile were identified as outliers and removed prior to statistical analysis. This criterion for excluding outliers was established to remove fish that failed to swim in a clip; without swimming a fish contributes no real movement data to the analysis. Features were compared between classes using a Student's *t*-test if the data were normally distributed or a Mann–Whitney *U*-test if the data were non-parametric. The investigator was not blinded to the genotype of zebrafish during video capture; however, when evolving classifiers, the extracted features, or PC values, fish were randomly organised into the three datasets – training, validation and test – and so the investigator was blinded to the genotype of fish when evolving a classifier.

### Training classification models

PCA was used to transform the multivariate time series of bend angles into a univariate time series suitable for classification. A type of EA called implicit context representation CGP has been effective at classifying PD movement in humans (Lones et al., 2017) by finding mathematical expressions that describe patterns of movement indicative of dyskinesia. This approach was used to train classifiers to discriminate the features of movement from PC2 time series data of *dj-1^−/^*^−^ fish from those of wild-type controls. Sliding window classifiers trained using the PC2 time series data generate symbolic mathematical expressions that describe discriminatory local patterns of movement within the data. The classifiers were trained using a stochastic optimisation algorithm and this was repeated multiple times (20) to generate multiple models. The features of movement and PC2 time series data from each recording were organised into three datasets: training, validation and test. To train classifiers with the features of movement, 60% of the recordings went into the training set, used to evaluate the effectiveness of classifiers during training. The remaining 40% of recordings were split equally between the validation and test sets. This split of the dataset proved the most effective to classify extracted features of movement. With a lower number of overall recordings used to train the CGP classifier, it made more sense to have a higher number in the training dataset. Aiming for a high number of clips to train the classifier allows it to recognise a common movement pattern. Whereas classifiers trained with the PC2 time series data had 50% of the recordings in the training set, 25% in the validation set and 25% in the test set, the sliding window classifier was evolved with a higher number of clips so more of the clips could go in the validation and test sets. This split of data was found best for evolving the most effective sliding window classifier. The validation set was used both for early stopping (i.e. to stop training when a model began to over-fit the data) and to select the most general classifier from the multiple models generated during training. The test set was used to get an unbiased measure of the selected model's discriminatory power. The tracking data were visually assessed to make sure they were of a high quality and the number of clips used in each class are shown in Tables S5 and S6. The sliding window classifiers were evolved using the AUC as a measure of fitness that is not sensitive to class imbalances.

## Supplementary Material

Supplementary information
