# Supplementary material for: Machine learning discriminates a movement disorder in a zebrafish model of Parkinson's disease
Source: Dis Model Mech. 2020 Oct 16;13(10):dmm045815. doi: 10.1242/dmm.045815 (PMC7578351; doi:10.1242/dmm.045815)
Supplement: Supplementary information [file dmm-13-045815-s1.pdf]

SUPPLEMENTARY DATA

Table S1

Primer sequences for qRT-PCR analysis (5' – 3').

Primer pairs that were designed based on previous publications: *dat, th* (Chen et al., 2012), *pitx3* (Sanchez-Simon et al., 2010) and *ef1a* (Fan et al., 2010).

Name	Forward primer	Reverse primer
dj-1	CGCACACAAACAGGGTCCATA	AAGCAAACCTCCAGGCAGAA
dat	CGTCACCAACGGTGGAATCTA	TGCCGATGGCCTCAATTAGTA
th	GACGGAAGATGATCGGAGACA	CCGCCATGTTCCGATTTCT
pitx3	GACAACAGTGACACAGAGAAGT	TGTCGGGATAACGGTTTCT
syn2a	CAAGGTTGTGAGGTCATTTAAGC	GTACTGGAGGCCGATGATTAAG
ef1a	TTGAGAAGAAAATCGGTGGTGCTG	GGAACGGTGTGATTGAGGGAAATTC
pkma	CCGCACACAGCACATGATTG	ACTGCATCTGAAGGGTCACG
vamp3	GAGCTGGATGATCGTGCTGA	ATGGCCCACATCTTGACGTT
brk	GCAGGTCTCGTTTAGCCACT	TTGTGACTCTGGCTTCGATGT
gpx3	ACCATAAACGGGACGCAGTT	AGTGCATTCAGTTCCACGTACT
gatm	TCCAGAGGAGTCAGGTTACGA	ATGTTGAAGGTGGCGTCGAT
abcf2a	CGGAACAGGCAAATCCATGC	GCTCTGGAATTGGCACCTCT
kcnk3b	AGGTCGTGTTGCTCCTGAAG	CATGGCCATAACCTATGGTTGTTA

Disease Models & Mechanisms • Supplementary information

Table S2a

Genes significantly up-regulated in dj-1$^{-/-}$ zebrafish brains

Genes that were up-regulated in the brains of *dj-1*$^{-/-}$ mutants (n = 3) when compared to their wild-type siblings (n = 3) at 16 wpf. Differentially expressed transcripts from the RNA-Seq analysis with a Q-value of <0.05 were considered significant. tpm = transcripts per million.

Gene Name	NCBI Transcript ID	Fold Change (log2)	q-value	Avg. WT tpm	Avg. dj-1-/- tpm
nfic	XM_009303916.3	8.22	0.01	0.00	0.70
mslna	XM_021474714.1	2.14	0.00	0.44	2.02
LOC110437990 (ncRNA)	XR_002455805.1	2.12	0.01	7.87	32.20
cldn19	NM_001017736.1	1.54	0.00	5.61	15.91
zgc:194629 (ncRNA)	NR_120380.1	1.01	0.00	55.40	110.82
mob2a	XM_005170681.4	0.88	0.00	9.92	18.14
adamts1	XM_021475923.1	0.79	0.02	2.68	4.60
kcnk3b	XM_694909.8	0.74	0.04	7.40	12.31
slc16a6b	XM_685174.9	0.65	0.03	9.49	14.80
abcf2a	NM_201315.2	0.52	0.03	54.02	77.35
prr5a	XM_005163058.4	0.49	0.04	19.10	26.79
strip2	XM_021475407.1	0.48	0.02	19.89	27.58
rnf144ab	XM_005160676.4	0.38	0.04	50.12	65.35
nr1d1	NM_205729.2	0.35	0.02	84.96	108.17
hsp90aa1.2	NM_001045073.1	0.34	0.03	127.68	161.99

Table S2b

Genes significantly down-regulated in dj1$^{-/-}$ zebrafish brains

Genes that were down-regulated in the brains of *dj-1*$^{-/-}$ mutants (n = 3) when compared to their wild-type siblings (n = 3) at 16 wpf. Differentially expressed transcripts from the RNA-Seq analysis with a Q-value of <0.05 were considered significant. tpm = transcripts per million.

Gene Name	NCBI Transcript ID	Fold Change (log2)	q-value	Avg. WT tpm	Avg. dj-1-/- tpm
grm2b	NM_001287547.1	-11.41	0.00	60.20	0.02
si:ch211-102c2.8 (ncRNA)	XM_021476476.1	-7.68	0.01	1.42	0.00
brk1	XM_005172903.4	-6.49	0.00	117.14	1.28
park7 (dj-1)	NM_001005938.1	-4.17	0.00	160.17	8.87
pkma	NM_199333.1	-3.62	0.00	496.18	38.60
vamp3	NM_001002073.1	-2.79	0.00	12.86	1.87
gatm	NM_199531.1	-2.56	0.00	33.80	5.57
her15.1	XM_003199526.5	-1.63	0.00	13.18	4.05
cldn19	XM_017358226.2	-1.36	0.00	38.59	15.09
kcnab2a	XM_009306291.3	-1.23	0.00	19.41	8.20
myef2	NM_001037423.1	-1.20	0.00	37.69	16.17
camkvb	NM_200450.1	-0.93	0.00	98.64	52.36
gpx3	NM_001137555.1	-0.76	0.04	33.62	19.65

Disease Models & Mechanisms • Supplementary information

Table S3
Gene set enrichment analysis (GSEA)

Hallmark gene sets enriched in the* dj-1$^{-/-}$ *zebrafish brain. A list of Hallmark gene sets found significantly enriched in the dj-1$^{-/-}$ zebrafish brain. The thresholds for significance were NES >1.5, p-value<0.05 and FDR-adjusted q-value<0.05. NES = normalized enrichment score.

Name	Gene set size	NES	p-val	FDR-adjusted q-val
G2M Checkpoint	125	2.11	0.00	0.00
Oxidative Phosphorylation	139	2.04	0.00	0.00
E2F Targets	131	1.87	0.00	0.00
Epithelial Mesenchymal Transition	98	1.82	0.00	0.01
Cholesterol Homeostasis	44	1.82	0.00	0.00
PI3K AKT MTOR Signaling	63	1.69	0.00	0.02
MYC Targets V1	135	1.62	0.00	0.03
MTORC1 Signaling	137	1.60	0.00	0.03
Mitotic Spindle	121	1.57	0.00	0.04
TGF beta Signaling	25	1.53	0.02	0.05
Androgen Response	64	1.53	0.01	0.04

Table S4

Selection and calculations of extracted features of movement

The features of movement being extracted and the previous research, investigating movement phenotypes in zebrafish, that inspired them. The principles used in this work are described in addition to the principles used in previous works.

**Feature**	**Based on**	**Stage in previous work**	**Principles in previous work**	**Principles used here**
Distance travelled	Keatinge et al., 2015	Adult	Total displacement of zebrafish over recording (taken side on)	Total displacement of zebrafish over recording (taken from above). Pythagoras theorem used to calculate distance travelled each second.
Velocity	Ingebretson and Masino, 2013	Larval	Centre of mass used to determine velocity in swimming episodes	Distance travelled divided by number of seconds spent swimming
Time spent moving	Godoy et al., 2015	Larval	Percentage of time spent moving over a 10-minute period	Percentage of time spent swimming at ≥ 5mm/s
Low/medium/high speed swimming	Keatinge et al., 2015	Adult	Low speed: <5cm/s<br>Medium speed: 5<x<7cm/s<br>High speed: >7cm/s	Low speed: 0.5cm<x<2cm/s<br>Medium speed: 2<x<4cm/s<br>High speed: >4cm/s
Mean swimming episode duration	Lambert et al., 2012	Larval	Speed of ≥ 3mm/s for swimming episode	Speed of ≥ 5mm/s for swimming episode
Tail bend amplitude	Budick and O'Malley, 2000	Larval	Angle between rostral and caudal tangents subtracted from 180°	Sum of angles along zebrafish trace at maximum tail bend (low/medium/high speed).
Tail beat frequency	Budick and O'Malley, 2000	Larval	Frequency of tail beat cycles in a second	Frequency of maximum tail bends per second (low/medium/high speed).

Disease Models & Mechanisms • Supplementary information

Table S5

Numbers used to evolve classifiers with features of movement

The number of mutant and age matched control clips used to evolve classifiers with features of movement for each mutant line. An equal number of clips needed to be present in each class as accuracy was used as the measure of fitness. Each clip also had to have a value present for the features calculated (e.g. low/medium/high speed features).

Mutants classified	No. of mutant clips	No. of age matched control clips
dj-1$^{-/-}$	30	30
pink1$^{-/-}$	37	37
dmd$^{ta222a/+}$	25	25

Table S6

Numbers used to evolve sliding window classifiers

The number of mutant and age matched control clips used to evolve sliding window classifiers for each mutant line. The sliding window classifiers used the area under the curve as a measure of fitness which is insensitive to class imbalances. This allowed each class to a have a different number of clips.

Mutants classified	No. of mutant clips	No. of age matched control clips
dj-1$^{-/-}$	36	64
pink1$^{-/-}$	39	44
dmd$^{ta222a/+}$	46	42

Fig. S1

Analysis of extracted features of movement in dj-1$^{-/-}$ zebrafish at 8 wpf

The features of movement compared between *dj-1*$^{-/-}$ and wild-type (WT) at 8 wpf including (A) distance travelled, (B) velocity, (C) percentage of time spent moving, (D) mean duration of a swimming episode, (E) tail beat frequency at low, medium and high swimming speeds, (F) tail bend amplitude at low, medium and high swimming speeds. * = P < 0.05, ** = P < 0.01, *** = P<0.001, ns = not significant.

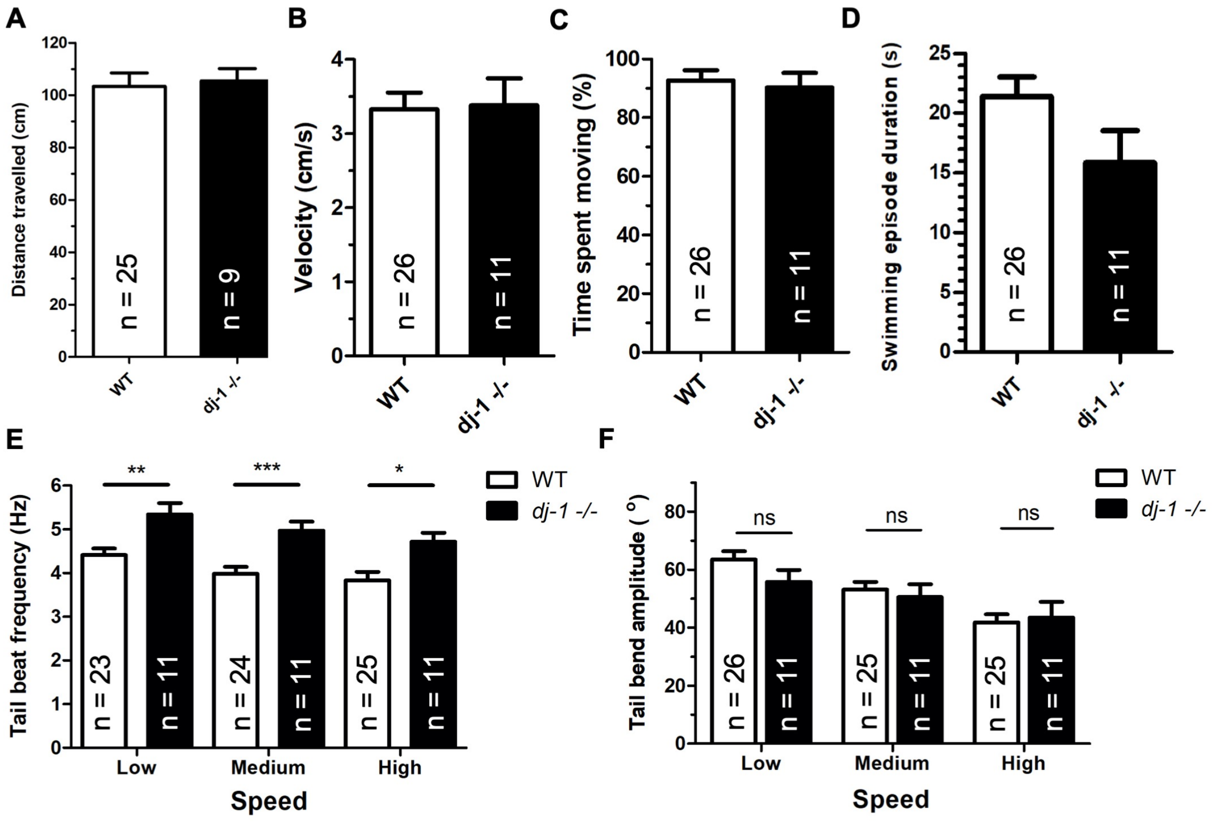

Disease Models & Mechanisms • Supplementary information

Fig. S2

Principle component analysis

The 5 principal components (PCs), generated from linear combinations of the 5 angles along the zebrafish spine, plot against time. The first PC (PC1) explains the most variation in the data, followed by PC2 and with each subsequent PC explaining less of the variation. Together all of the PCs explain 100% of the variation within the tail bend angle data. PC1 captured only broad movements whilst PC2 retained some high frequency movements.

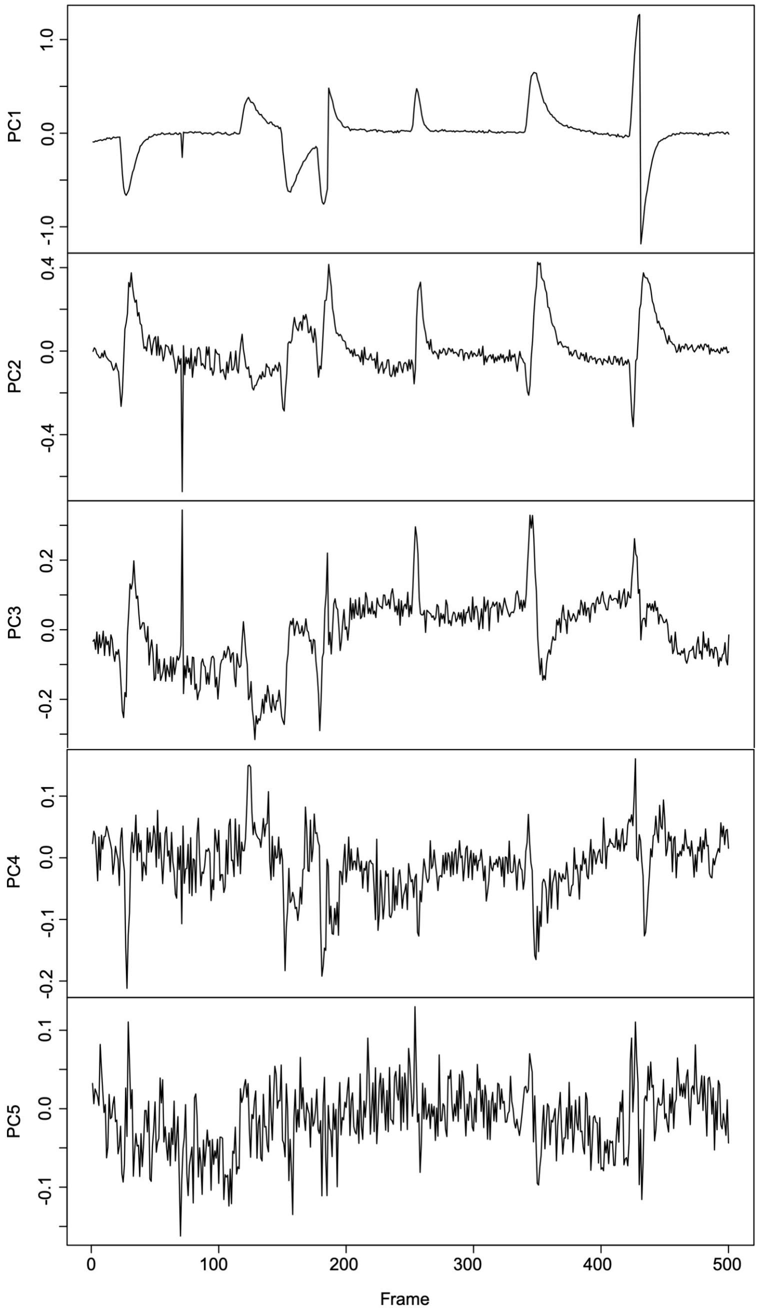

Disease Models & Mechanisms • Supplementary information

Fig. S3

Scores of the dmd$^{ta222a/+}$ sliding window classifiers

Training and test scores (AUCs) from the 20 sliding window classifiers evolved using the PC2 time series data from the 20 folds of data set.

Run	Training score	Test score
1	0.7318	0.3833
2	0.7227	0.3166
3	0.8636	0.45
4	0.8137	0.6334
5	0.8591	0.3834
6	0.8046	0.5334
7	0.7591	0.5334
8	0.8409	0.5833
9	0.8137	0.5167
10	0.7773	0.3167
11	0.7636	0.4166
12	0.7228	0.35
13	0.7409	0.4833
14	0.7727	0.25
15	0.7772	0.5166
16	0.7454	0.4166
17	0.7455	0.3167
18	0.7818	0.45
19	0.7682	0.3834
20	0.7182	0.3334
**Mean**	0.77614	0.42834

Disease Models & Mechanisms • Supplementary information
